# Verwendung der intraaortalen Ballonpumpe zur Verbesserung der zerebralen Sauerstoffsättigung nach Reanimation bei einem herzchirurgischen Eingriff

**DOI:** 10.1007/s00101-023-01351-8

**Published:** 2023-10-19

**Authors:** Anja Funk, Erich Kilger, Polyxeni Vlachea, Dominik J. Höchter

**Affiliations:** 1 Klinik für Anaesthesiologie, Klinikum der Ludwig-Maximilians-Universität München, Marchioninistr. 15, 81377 München, Deutschland; 2 Klinik für Herzchirurgie, LMU Klinikum, Klinikum der Ludwig-Maximilians-Universität, Marchioninistr. 15, 81377 München, Deutschland

**Keywords:** IABP, NIRS, Kardioanästhesie, Zerebrale Sättigung, Mechanische Assist Devices, IABP, NIRS, Cardiothoracic anesthesiology, Cerebral oxygen saturation, Mechanical assist devices

## Falldarstellung

### Anamnese

Bei einer 77-jährigen Patientin kam es im Rahmen eines Nicht-ST-Hebungsinfarktes zur kardialen Dekompensation. Eine weiterführende Diagnostik ergab eine schwergradige, koronare Dreigefäßerkrankung mit mittel- bis hochgradig eingeschränkter linksventrikulärer Pumpfunktion. Die transthorakale echokardiographische Untersuchung wies zusätzlich eine hochgradige Aortenklappenstenose mit einer Klappenöffnungsfläche von 0,7 cm^2^ und einem maximalen Gradienten von 82 mm Hg auf. Nebenbefundlich fand man eine beidseitige Stenose der Aa. carotides (50–60 %). Die Indikation zum konventionellen Aortenklappenersatz mit zusätzlicher Anlage von aortokoronaren Bypässen wurde interdisziplinär gestellt.

### Befund

Die Patientin war nach Standard auf dem Rücken gelagert, der Oberkörper leicht erhöht, der Kopf lag auf einem Kopfring. Vor der Narkoseeinleitung wurden Blutdruckwerte von 130/80 mm Hg gemessen. Nach der Einleitung der Allgemeinanästhesie mit 0,8 µg/kgKG Sufentanil, 0,8 mg/kgKG Propofol, 0,8 mg/kgKG Ketamin (Razemat) und 0,8 mg/kgKG Rocuronium waren Dosierungen von Norepinephrin (0,2 μg/kgKG und min) nötig, um einen mittleren arteriellen Blutdruck > 65 mm Hg aufrechtzuerhalten. Die Patientin hatte einen präoperativen Hämoglobinwert von 12,6 g/dl und einen Horowitz-Index von 204 nach der Intubation (unter einer Beatmung mit einer F_I_O_2_ von 0,48). Die Anlagen von ZVK und venöser 8,5-F-Schleuse erfolgten komplikationslos ultraschallgesteuert in die linke V. jugularis interna. Die intraoperativ durchgeführte transösophageale Echokardiographie (TEE) zeigte erneut eine mittel- bis hochgradig eingeschränkte linksventrikuläre Pumpfunktion und keine neu aufgetretenen Wandbewegungsstörungen. Zeitgleich imponierten bilateral niedrige NIRS-Werte. Es wurde ein INVOS™-Cerebral/Somatic-Oxymeter (Healthcare 21 Group, Cork, Irland) verwendet, das auf beiden Stirnhälften eine regionale Sauerstoffsättigung (rS_c_O_2_) von 28 % anzeigte. Eine Verbesserung der Hämodynamik auf der Basis von Inotropiesteigerung zur Erhöhung der rS_c_O_2_ wurde angestrebt.

Noch vor dem Hautschnitt kam es im OP zu Kammerflimmern. Die Patientin wurde über einen Zeitraum von 1–2 min mechanisch reanimiert. Ohne Defibrillation konvertierte das Kammerflimmern in einen Sinusrhythmus.

### Diagnose

Hämodynamische Instabilität bei Myokardischämie im Rahmen einer koronaren Herzerkrankung mit eingeschränkter linksventrikulärer Pumpfunktion und Aortenklappenstenose.

### Therapie und Verlauf

Aufgrund dieser Gesamtkonstellation wurde die Entscheidung zur Implantation einer intraaortalen Ballonpumpe (IABP) gefällt.

Über eine 8‑F-Schleuse wurde der intraaortale Ballonkatheter Sensation Plus 8 Fr 50 cc über die rechte A. femoralis gelegt und mit der Cardiosave-Konsole (Getinge, Göteborg, Schweden) verbunden. Die IABP wurde mit einer Unterstützung von 1:1 gestartet.

**Figure 1:**
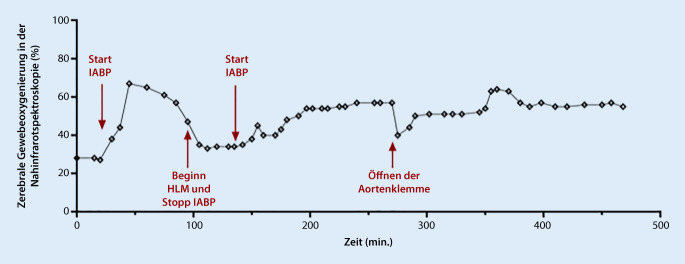

Die rS_c_O_2_ stieg in kurzer Zeit von < 30 % auf Werte zwischen 57 und 67 % auf beiden Kopfseiten (Abb. 1). Nach der Präparation der Gefäß-Grafts wurde die extrakorporale Zirkulation etabliert. Mit Beginn der Herz-Lungen-Maschine (HLM) stellten wir die IABP aufgrund von Alarmierungen bei fehlenden Druck- und EKG-Triggern in den Standby-Modus. Die NIRS-Werte fielen daraufhin auf Werte zwischen rS_c_O_2_ 33 und 35 %. Nach Ausnutzen aller verbessernden Maßnahmen wie Erhöhung der F_I_O_2_ auf 1,0, Normalisierung des mittleren arteriellen Blutdrucks auf Werte > 65 mm Hg, Vermeidung einer Hyperventilation (etCO_2_ zwischen 38 und 46 mm Hg, p_a_CO_2_ 39–50 mm Hg), Lagekontrolle der HLM-Kanülen, Erhöhung des HLM-Flusses von 100 % auf 120 % nahmen wir die IABP erneut in Betrieb und wählten den internen Modus mit 80/min. Ein erneuter kontinuierlicher Anstieg der NIRS-Werte konnte verzeichnet werden (Abb. 1).

Die Gefäßanastomosen wurden etabliert, danach erfolgte der Ersatz der Aortenklappe mittels einer biologischen Aortenklappenprothese. Der initiale Rhythmus nach dem Eröffnen der Aortenklemme war erneut Kammerflimmern. Ohne Defibrillation konvertierte der Rhythmus innerhalb kurzer Zeit in einen Kammerersatzrhythmus bei AV-Block III°. In dieser Phase nach der Eröffnung zeigte sich ein erneuter Abfall der NIRS-Werte beidseits auf 40 %. Epikardiale Schrittmacherelektroden wurden aufgenäht und das Herz im D00-Modus mit einer Frequenz von 80/min stimuliert. Die IABP wurde in den druckgetriggerten Modus umgestellt. Die NIRS-Werte stiegen wieder auf 57 % (Abb. 1). Während der verlängerten Reperfusion unterstützte die IABP kontinuierlich und die NIRS-Werte lagen bei 60 %. Der weitere Operationsverlauf war komplikationslos, und die Patientin konnte unter moderater Katecholamintherapie mit einliegender IABP auf die Intensivstation verlegt werden.

Die Patientin konnte bei anfänglich eingeschränktem pulmonalem Gasaustausch am ersten postoperativen Tag extubiert werden. Sie war bereits am ersten postoperativen Tag 4fach orientiert (zu Person, Zeit, Ort und Situation). Es zeigten sich keine neurologischen Defizite. Die IABP wurde 48 h nach dem Eingriff bei anhaltendem Cardiac Index > 2,5 l/min und m^2^KOF entfernt. Die Patientin wurde wegen prolongiertem Atemtrainings mit NIV-Therapie am vierten postoperativen Tag von der Intensivstation auf die Normalstation verlegt und am 12. postoperativen Tag in gutem Allgemeinzustand aus dem Krankenhaus entlassen.

## Diskussion

Herzchirurgische Eingriffe sind mit einem erhöhten Risiko für neurologische Komplikationen assoziiert. Die wissenschaftlichen Arbeitskreise Kardioanästhesie der Deutschen Gesellschaft für Anästhesiologie und Intensivmedizin (DGAI) sowie die Deutsche Gesellschaft für Thorax‑, Herz- und Gefäßchirurgie (DGTHG) empfehlen ein Neuromonitoring während herzchirurgischer Eingriffe [1].

Diese Empfehlungen beinhalten die Messung der „Zerebraloxymetrie“, deren Grundlage die Nahinfrarotspektroskopie (NIRS) ist. Die resultierenden Messwerte werden als regionale zerebrale Sauerstoffsättigung (rS_c_O_2_) bezeichnet.

Die NIRS misst die rS_c_O_2_ indirekt. Das intrakranielle Blutvolumen besteht zu 25–30 % aus arteriellen und zu 70–75 % aus venösen Komponenten. Im Gegensatz zur Pulsoxymetrie beurteilt die NIRS hauptsächlich den nichtpulsatilen venösen Anteil des Blutes und erlaubt eine kontinuierliche und nichtinvasive Echtzeitmessung der rS_c_O_2_ innerhalb eines Bereichs des frontalen Kortex [2].

Die DGAI empfiehlt, eine NIRS-Überwachung bei Korrekturoperationen angeborener Herzfehler, Operationen im Kindesalter sowie bei Operationen an der thorakalen Aorta im Erwachsenenalter einzusetzen [1]. Als „optionale Empfehlung“ kann eine NIRS-Überwachung bei Vorliegen eines erhöhten Patienten- oder Operationsrisikos verwendet werden. Dazu zählen beispielsweise Karotisstenosen, wie sie in diesem Fall vorlagen. Eine Studie von Colak et al. [3] zeigte, dass Patienten, welche eine intraoperative NIRS-Überwachung während ihrer Bypass Operation erhielten, ein signifikant besseres neurologisches Outcome hatten, als Patienten ohne Neuromonitoring. Die Arbeit demonstrierte, dass ein prolongierter Abfall der rS_c_O_2_ ein Prädiktor für eine kognitive Dysfunktion ist [3].

Auch an unserer Klinik hat das NIRS-Monitoring zur Detektion eines rS_c_O_2_-Abfalls während herzchirurgischer Eingriffe einen hohen Stellenwert. Leider erfolgte aus Kapazitätsgründen keine Messung am wachen Patienten in der Einleitung, um einen Ausgangswert zu bestimmen, was bei diesem Fall sehr hilfreich gewesen wäre.

Bei NIRS-Abfall soll der Handlungsalgorithmus nach Denault et al. befolgt werden [4], der folgende Punkte umfasst: Sollte der arterielle Blutdruck vermindert sein, wird dieser normalisiert; eine Hyperventilation wird vermieden; eine mögliche Anämie sollte im Rahmen des Patient Blood Management korrigiert werden; der zerebrale Sauerstoffverbrauch wird mittels Narkosevertiefung reduziert, und die Lage vorhandener HLM-Kanülen wird durch den Operateur und den Anästhesisten mittels TEE überprüft. Diese Maßnahmen zeigten beim dargestellten Fall keine Verbesserung der rS_c_O_2_. Erst nach der Implementierung der IABP kam es zu einem deutlichen Anstieg der rS_c_O_2_.

Die IABP wurde Ende der 60er-Jahre des letzten Jahrhunderts in die Klinik eingeführt und ist somit das älteste und am einfachsten einzusetzende mechanische Kreislaufunterstützungssystem. Der Nutzen der IABP liegt darin, dass die Ballonpumpe die Nachlast während der Systole reduziert, den koronaren und zerebralen Perfusionsdruck während der Diastole anhebt und somit den kardialen Auswurf erhöht, v. a. bei Patienten mit Myokardischämie. In diesem Fall dürften die diastolische Augmentation des Blutflusses zu einer Verbesserung der koronaren und systemischen Durchblutung und das Ablassen des Ballons in der nächsten Systole zu einer Nachlastsenkung und damit Entlastung des Myokards mit Steigerung des Herzzeitvolumens geführt haben. Sowohl die Entlastung des Myokards als auch die verbesserte diastolische Perfusion haben dabei positive Effekte auf den Sauerstoffbedarf des Herzen und damit auch auf die zentralvenöse Sauerstoffsättigung (S_zv_O_2_). Dennoch muss aber erwähnt werden, dass es auch Studien gibt, die zeigten, dass nicht jede Änderung des rS_c_O_2_ mit einer Änderung der S_zv_O_2_ korreliert [5].

Die IABP wurde in der deutschen Kardiologie aufgrund der neutralen Ergebnisse einer mit zahlreichen Limitationen [6], u. a. fehlender statistischer Power, behafteten Studie bei Patienten mit myokardinfarktassoziiertem kardiogenen Schock [7] von wesentlich invasiveren Verfahren, die mit einer höheren Sterblichkeit und Komplikationsrate behaftet sind [8], wie Impella® oder ECLS verdrängt. Außerhalb von Deutschland stellt die IABP aber unverändert das mechanische Kreislaufunterstützungsverfahren der ersten Wahl bei herzchirurgischen Patienten dar und hat entsprechend in Leitlinien oder Expertenempfehlungen einen hohen Empfehlungsgrad [9, 10].

Die S3-Leitlinie zum Einsatz der IABP in der Herzchirurgie, die sich aktuell in Überarbeitung befindet, spricht sich für eine präoperative Implantation einer IABP bei hämodynamisch stabilen Hochrisikopatienten aus (Evidenzgrad Ib). Mit dem höchsten Evidenzgrad (Ia) wird empfohlen, eine präoperativ etablierte IABP während des kardiochirurgischen Eingriffs weiterlaufen zu lassen, um den nichtpulsatilen Fluss der HLM in einen pulsatilen Fluss umzuwandeln.

Studien zeigten einen signifikant positiven Effekt des IABP-induzierten pulsatilen Flusses auf die Nierenfunktion, die Splanchnikusperfusion und die Lungenfunktion (Compliance, Oxygenierungsindex) sowie auf das Gerinnungssystem (Fibrinogen, Thrombozyten, Hämatokrit etc.) und die Endothelaktivierung (VEGF, MCP-1) [11].

Der Einfluss einer IABP auf die zerebrale Perfusion wird in der Literatur kontrovers beschrieben. In den folgenden Studien wurde die zerebrale Perfusion mittels transkranieller Dopplersonographie gemessen. Pfluecke et al. zeigten, dass die Verwendung der IABP bei Patienten mit akuter kardialer Dekompensation den zerebralen Blutfluss verbesserte [12]. 2014 untersuchten Yang et al. 12 Patienten, die eine IABP und eine extrakorporale Membranoxygenierung (ECMO) nach herzchirurgischen Eingriffen benötigten, und berichteten, dass Änderungen im zerebralen Blutfluss abhängig von der IABP waren [13].

Nur wenige Studien widmen sich der Thematik, ob die durch die IABP verursachte pulsatile Perfusion während einer Operation an der HLM die zerebrale Sauerstoffsättigung verbessert. Eine Studie von Kawahara et al. beschäftigte sich mit dieser Fragestellung und untersuchte 11 Patienten mit intraoperativ laufender IABP an der HLM und 11 Patienten ohne IABP. Es gab keinen Unterschied der NIRS-Werte in den beiden Gruppen [14]. Dies könnte daran gelegen haben, dass die eingeschlossenen Patienten weder renale, pulmonale, neuro- oder neurovaskuläre Erkrankungen hatten.

Eine weitere Überlegung wäre, die IABP ohne Schleuse zu implantieren. Sehr aktuelle Daten zeigten, dass eine „schleusenlose“ Implantation mit weniger Komplikationen und einem besseren Outcome verbunden ist [15].

Die zeitliche Anlage der IABP, die intraoperative Pulsation und die NIRS-Messung waren ein wichtiger Bestandteil des Managements bei dem hier beschriebenen Fall. Die Patientin im dargestellten Kasus hatte mit den beidseitigen Karotisstenosen Risikofaktoren. Die Entscheidung, eine IABP anzulegen, war durch die zunehmend eingeschränkte linksventrikuläre Pumpfunktion der Patientin, den steigenden Katecholaminbedarf und der Reanimation begründet. Sinnvoll und leitliniengerecht wäre es gewesen, die IABP bereits vor der Narkoseeinleitung zu implementieren.

Die gemessene rS_c_O_2_ zeigte einen eindeutigen Anstieg, nachdem die IABP während der HLM in Gang gesetzt wurde. Es ist naheliegend, dass der pulsatile Fluss der IABP zur verbesserten Hirndurchblutung beigetragen hat. Des Weiteren könnte die Veränderung der rS_c_O_2_ auch ein Ausdruck einer verbesserten systemischen Perfusion sein. Dies zu differenzieren, wäre nur über ein hämodynamisches Monitoring beispielsweise mittels Pulmonalarterienkatheter und kontinuierlicher Überwachung der S_zv_O_2_ möglich und bei derartigen Risikopatienten sinnvoll gewesen. Intraoperativ entschieden wir uns nach dem Kammerflimmern dagegen, einen Pulmonalarterienkatheter einzuschwemmen. Wir untersuchten die Hämodynamik während der Operation mittels TEE; unmittelbar postoperativ erfolgte die Etablierung eines PiCCO-Messverfahrens, wobei wir zur Einschätzung des postoperativen HZV unter IABP-Therapie nur die Thermodilutionswerte und nicht die Pulskonturwerte verwendeten.

## Fazit für die Praxis

Diese Kasuistik deutet darauf hin, dass die intraoperative Anwendung der IABP während einer herzchirurgischen Operation mit HLM speziell bei Risikopatienten durch den pulsatilen Fluss zu einer Verbesserung der zerebralen Oxygenierung beitragen kann. Es gibt kaum Studien, die sich mit dieser Thematik beschäftigen und die die NIRS-Messung als Indikator der zerebralen Oxygenierung nutzten.

## References

[1] Deutsche Gesellschaft für Anästhesiologie und Intensivmedizin, Schweizerische Gesellschaft für Anästhesiologie und Reanimation, Deutsche Gesellschaft für Thorax- Herz-und Gefäßchirurgie (2014). Neuromonitoring in der kardioanästhesie. Z Herz- Thorax- Gefäßchir.

[2] Bolkenius D, Dumps C, Rupprecht B (2021). Near-infrared spectroscopy : technique, development, current use and perspectives. Anaesthesist.

[3] Colak Z, Borojevic M, Bogovic A, Ivancan V, Biocina B, Majeric-Kogler V (2015). Influence of intraoperative cerebral oximetry monitoring on neurocognitive function after coronary artery bypass surgery: a randomized, prospective study. Eur J Cardiothorac Surg.

[4] Denault A, Deschamps A, Murkin JM (2007). A proposed algorithm for the intraoperative use of cerebral near-infrared spectroscopy. Semin Cardiothorac Vasc Anesth.

[5] Schmidt C, Heringlake M, Kellner P, Berggreen AE, Maurer H, Brandt S (2018). The effects of systemic oxygenation on cerebral oxygen saturation and its relationship to mixed venous oxygen saturation: a prospective observational study comparison of the INVOS and foresight elite cerebral oximeters. Can J Anaesth.

[6] Heringlake M, Sander M, Ender J (2023). Klinischer stellenwert sowie risiken bei anwendung und ausfall einer intraaortalen ballonpumpe (IABP). Eine Stellungnahme des wissenschaftlichen arbeitskreises kardioanästhesie der deutschen gesellschaft für anästhesiologie und Intensivmedizin e. V. (DGAI). Anästhesiol Intensivmed.

[7] Thiele H, Zeymer U, Neumann FJ, Ferenc M, Olbrich HG, Hausleiter J (2012). Intraaortic balloon support for myocardial infarction with cardiogenic shock. N Engl J Med.

[8] Dhruva SS, Ross JS, Mortazavi BJ, Hurley NC, Krumholz HM, Curtis JP (2020). Association of use of an intravascular microaxial left ventricular assist device vs intra-aortic balloon pump with in-hospital mortality and major bleeding among patients with acute myocardial infarction complicated by cardiogenic shock. JAMA.

[9] Pilarczyk K, Bauer A, Boening A, von der Brelie M, Eichler I, Gohrbandt B (2015). S3-guideline: recommendations for intra-aortic balloon pumping in cardiac surgery. Thorac Cardiovasc Surg.

[10] Bakaeen FG, Gaudino M, Whitman G, Doenst T, Ruel M, Taggart DP (2021). The american association for thoracic surgery expert consensus document: coronary artery bypass grafting in patients with ischemic cardiomyopathy and heart failure. J Thorac Cardiovasc Surg.

[11] Serraino GF, Marsico R, Musolino G, Ventura V, Gulletta E, Santè P (2012). Pulsatile cardiopulmonary bypass with intra-aortic balloon pump improves organ function and reduces endothelial activation. Circ J.

[12] Pfluecke C, Christoph M, Kolschmann S, Tarnowski D, Forkmann M, Jellinghaus S (2014). Intra-aortic balloon pump (IABP) counterpulsation improves cerebral perfusion in patients with decreased left ventricular function. Perfusion.

[13] Yang F, Jia ZS, Xing JL, Wang Z, Liu Y, Hao X (2014). Effects of intra-aortic balloon pump on cerebral blood flow during peripheral venoarterial extracorporeal membrane oxygenation support. J Transl Med.

[14] Kawahara F, Kadoi Y, Saito S, Yoshikawa D, Goto F, Fujita N (1999). Balloon pump-induced pulsatile perfusion during cardiopulmonary bypass does not improve brain oxygenation. J Thorac Cardiovasc Surg.

[15] Heuts S, Lorusso R, di Mauro M, Jiritano F, Scrofani R, Antona C (2023). Sheathless versus sheathed intra-aortic balloon pump implantation in patients undergoing cardiac surgery. Am J Cardiol.

